# Optimization of the Surface Structure on Black Silicon for Surface Passivation

**DOI:** 10.1186/s11671-017-1910-6

**Published:** 2017-03-16

**Authors:** Xiaojie Jia, Chunlan Zhou, Wenjing Wang

**Affiliations:** 1 The Key Laboratory of Solar Thermal Energy and Photovoltaic System, Institute of Electrical Engineering, Chinese Academy of Science (CAS), Beijing, China; 2 University of Chinese Academy of Sciences (UCAS), Beijing, China

**Keywords:** Black silicon, MCCE, Passivation, Illumination

## Abstract

Black silicon shows excellent anti-reflection and thus is extremely useful for photovoltaic applications. However, its high surface recombination velocity limits the efficiency of solar cells. In this paper, the effective minority carrier lifetime of black silicon is improved by optimizing metal-catalyzed chemical etching (MCCE) method, using an Al_2_O_3_ thin film deposited by atomic layer deposition (ALD) as a passivation layer. Using the spray method to eliminate the impact on the rear side, single-side black silicon was obtained on n-type solar grade silicon wafers. Post-etch treatment with NH_4_OH/H_2_O_2_/H_2_O mixed solution not only smoothes the surface but also increases the effective minority lifetime from 161 μs of as-prepared wafer to 333 μs after cleaning. Moreover, adding illumination during the etching process results in an improvement in both the numerical value and the uniformity of the effective minority carrier lifetime.

## Background

Black silicon (b-Si), also called nanostructured surface silicon, shows much lower light reflection losses than conventional random pyramids [1] and thus is an extremely promising material for photovoltaic applications [2]. Up to now, there are three kinds of way to fabricate b-Si: laser texturing [3], reactive ion etching (RIE) [4], and metal-catalyzed chemical etching (MCCE) [5]. Among these, RIE and MCCE techniques have obtained high expectations. However, from the point of view of industry matching and cost-effectiveness, MCCE method is much more suitable for large-scale production, since the conventional texturing process is also based on wet chemical etching [6].

Ye et al. reported a novel nanoscale pseudo-pyramid texture formed by a MCCE technique and an additional NaOH solution treatment [7]: step 1, wafers were dipped into AgNO_3_/HF mixed solution to grow Ag nanoparticles on surface; step 2, wafers were steeped in the HF/H_2_O_2_ mixed solution to do the MCCE process; step 3, wafers were immersed in dense HNO_3_ to remove Ag ions; and step 4, a NaOH solution treatment to reduce the surface area and convert the microporous layer into a nanoscale pseudo-pyramid texture. This method has been proven as an efficient way to obtain high-efficiency black multi-crystalline (mc) silicon solar cells. Nevertheless, there still is a room for optimization.

Firstly, etching process increases the surface recombination, so it is necessary that only the front side of the wafer is etched while the rear side gets protected. Normally, the rear side can be protected by a mask before step 1 and the mask will be washed off after step 4 [8]. However, the preparation of the mask increases the process steps. Therefore, in our experiments, the spray method was used to deposit the AgNO_3_/HF mixed solution onto the wafer surface. Since the spray method is applied to only one side of the sample, no preparation of the mask is necessary. Secondly, NaOH solution used in step 4 needs to be washed with HCl solution to remove Na^+^. If NH_4_OH/H_2_O_2_/H_2_O in the standard RCA cleaning is used, not only the alkaline treatment can be achieved but also the cleaning step can be omitted. Thirdly, in order to improve the reaction rate and the uniformity of the etching process, it is possible to refer to the photo-induced plating (LIP) technique by adding illumination during the etching process [9]. In the absence of illumination, because of the electrochemical potential of the oxidant (H_2_O_2_) being much more positive than the valence band of Si, silver ions act as a catalyst to rapidly reduce H_2_O_2_ and produce copious number of holes injected into the valence band of Si [10]. If a certain amount of illumination is provided, the number of photo-generated holes can be comparable with or higher than the holes obtained by reducing H_2_O_2_, thus accelerating the etching rate [8].

In order to improve the efficiency of solar cells, b-Si needs excellent surface passivation [11]. Although a wide variety of films can be selected, such as SiN_x_ [12] and SiO_2_ [13], the Al_2_O_3_ thin film deposited by atomic layer deposition (ALD) is the best choice for passivating b-Si [14]. On the one hand, ALD, which has a conformal growth on surface with high aspect ratio features [15] and pinhole-free nature [16], is the natural choice for the coating of nanostructured surfaces. On the other hand, Al_2_O_3_ is a suitable surface passivation material for silicon-based photovoltaic applications [17]. Al_2_O_3_ layer not only enables excellent chemical passivation due to strong coordination of Si and O [18] and selective hydrogenation leading to a low interface state density after annealing [19], but also, it provides a strong field effect passivation through a high concentration of fixed negative charges [20] that leads to repulsion of charge carriers from the entire surface [21].

## Methods

**Fig. 1:**
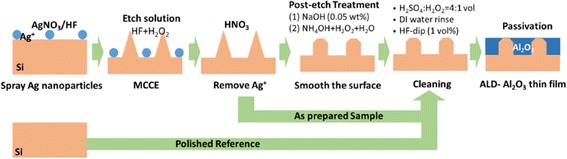
Schematics of the fabrication flow for b-Si using optimized MCCE method. Use polished wafer as a reference

The experiments were performed on double-side polished n-type solar grade crystalline silicon wafers (COMTEC Co. Ltd.) of resistivity 1.7 ~ 13 Ω · cm. Single-side black silicon was fabricated using MCCE method. After spraying the AgNO_3_/HF mixed solution (AgNO_3_ in 0.001 mol/L, HF in 0.24 mol/L) on the front side of wafer to deposit silver nanoparticles, the wafers were rinsed by deionized (DI) water. Then, steep wafers into the HF/H_2_O_2_ mixed solution for a few minutes to do the MCCE process. According to different concentration ratio of HF and H_2_O_2_, the chemical etching solution is divided into two kinds: etch solution A, HF (2.11 mol/L)/H_2_O_2_ (0.36 mol/L), and etch solution B, HF (0.42 mol/L)/H_2_O_2_ (0.18 mol/L). After that, the wafers were immersed into concentrated HNO_3_ solution for 10 min and then rinsed by DI water to remove silver nanoparticles. In order to smooth the surface, alkaline solutions were used as post-etch treatment. Apart from the NaOH solution (0.05 wt%), NH_4_OH/H_2_O_2_/H_2_O mixed solution was also used, and the NH_4_OH content was adjusted (NH_4_OH:H_2_O_2_:H_2_O = 0.2:1:5 (J), 0.5:1:5 (K), and 1.0:1:5 (L) (vol)). Schematics of the fabrication flow for the b-Si are shown in Fig. 1, using polished wafer as a reference.

After the post-etch treatments, samples were sequentially washed with H_2_SO_4_/H_2_O_2_ mixed solution (H_2_SO_4_:H_2_O_2_ = 4:1 vol) at 80 °C for 10 min, DI water rinse and HF-dip (1 vol%) at room temperature for 60 s. Al_2_O_3_ thin film was deposited on both sides of wafers by plasma-enhanced atomic layer deposition (PE-ALD) at 200 °C. Trimethylaluminium (TMA, Al_2_(CH_3_)_6_) was chosen as aluminum source and plasma oxygen as the oxidant. Polished samples (without b-Si) were also processed as references. The thickness of the thin film was about 10 nm, measured by ellipsometry on polished wafers. The passivation was activated by post-annealing at the temperature of 450 °C for 10 min in ambient air.

In order to evaluate the anti-reflection quality of b-Si, the reflection spectrum was measured in the range of 300 to 1200 nm. The AM1.5G-weighted reflectance (*R*_*W*_) was calculated by Equation (1)

1 $$ {R}_W=\frac{{\displaystyle {\int}_{300}^{1200} R\left(\lambda \right){F}_{\mathrm{Ph}}\left(\lambda \right)\; d\lambda}}{{\displaystyle {\int}_{300}^{1200}{F}_{\mathrm{Ph}}\left(\lambda \right)\; d\lambda}} $$

where *R*(*λ*) is the measured reflectance and *F*_Ph_(*λ*) is the incident photon flux of the AM1.5G spectrum [22]. Effective minority carrier lifetime maps of samples were measured after annealing using the microwave-detected photoconductive decay (μ-PCD) (WT-2000, Semilab). The surface morphology of b-Si was analyzed using high resolution scanning electron microscope (SEM) (Orion NanoFab, Zeiss), in which focused ion beam (FIB) was used to create the cross section.

## Results and Discussions

### Effect of the Etch Solution

**Fig. 2:**
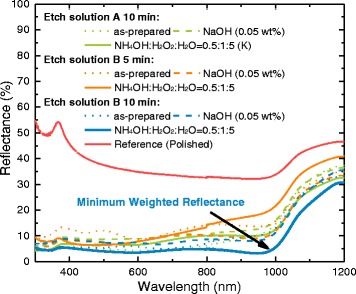
Reflectance spectrums of samples etched by solutions A and B with different post-etch treatments, using polished wafer as a reference

The reflectance spectrums of bare silicon etched by solutions A and B with different post-etch treatments are shown in Fig. 2 and the AM1.5G-weighted reflectance in Table 1, using polished wafer as a reference. Black silicon achieves a reflectance below 10% in the whole visible spectrum as well as in the near UV and near IR regions. As a comparison, the reflectance of polished wafer without anti-reflection coating varies between 30 and 50% in the same wavelength range.

**Table 1:** The AM1.5G-weighted reflectance of samples etched by solutions A and B with different post-etch treatments, using polished wafer as a reference

Weighted reflectance (%)			
Post-etch treatment	Etch solution		
	A 10 min	B 5 min	B 10 min
As-prepared	8.4	13.6	8.6
NaOH (0.05 wt%)	12.0	10.7	9.8
NH_4_OH:H_2_O_2_:H_2_O = 0.5:1:5	9.9	13.0	6.0
Reference (polished)	36.5		

For as-prepared samples, the volume ratio of HF/H_2_O_2_ in etch solutions A and B did not significantly affect the weighted reflectance at the same etching time. In etching for 10 min, samples show weighted reflectance of 8.4 and 8.6%, respectively, which are basically the same. Compared with the sample etched for less time, the value of the weighted reflectance decreases as the etching time increases.

However, samples etched by different solution shows different performance after the post-etch treatment, taking samples with an etching time of 10 min as example. In post-etch treated by NaOH mixed solution, all samples show an increasing weighted reflectance, but samples etched by solution A increase more. In post-etch treated by NH_4_OH/H_2_O_2_/H_2_O mixed solution, sample etched by solution A still shows an increasing of weighted reflectance. However, the weighted reflectance of sample etched by solution B decreased after NH_4_OH/H_2_O_2_/H_2_O post-etching treatment and, finally, obtained the minimum value of weighted reflectance of 6%. This value is evidently better than the ones obtained in industrial alkaline-textured single crystalline silicon wafers or even the inverted pyramids of 15% without antireflection coating [23].

**Fig. 3:**
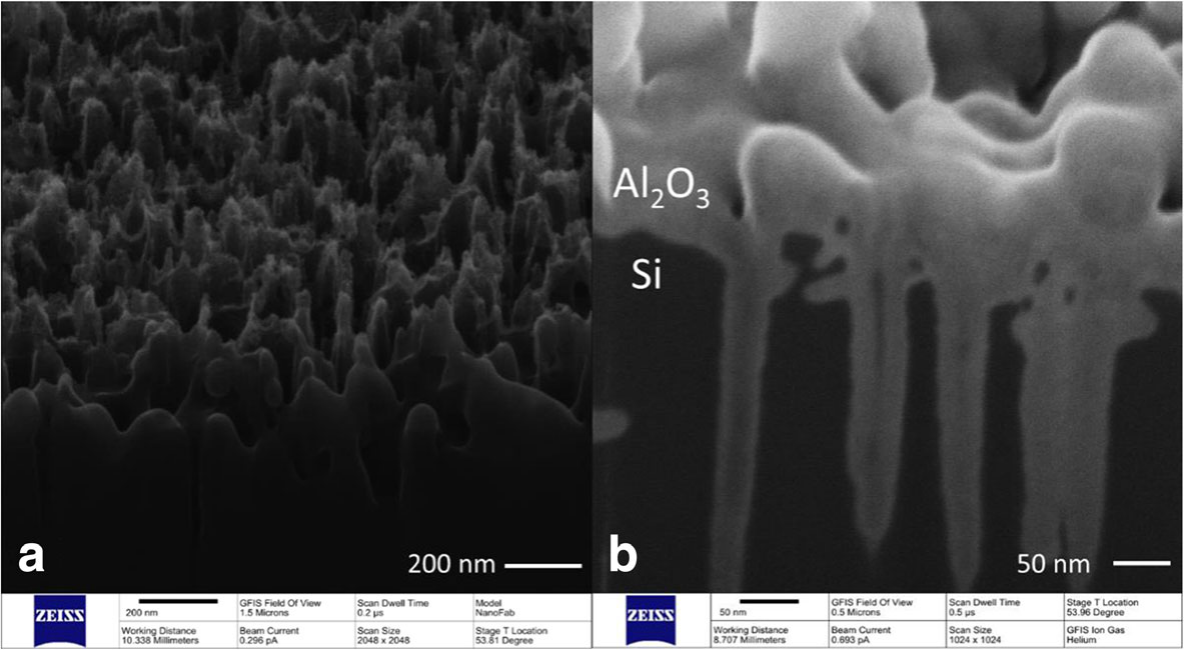
The cross-sectional SEM picture of b-Si. **a** Samples etched by HF/H_2_O_2_ mixed solution without Al_2_O_3_ coating. **b** Samples etched by HF/H_2_O_2_ mixed solution with 10 nm Al_2_O_3_ coating

In order to improve the surface passivation of b-Si, a 10-nm Al_2_O_3_ thin film coating was deposited by PE-ALD on both sides of wafers. Figure 3 shows the high resolution cross-sectional SEM picture of b-Si with and without Al_2_O_3_ coating. Samples were etched by solution B and without any post-etch treatment after Ag^+^ removal. Compared with the samples without coating, a highly conformal Al_2_O_3_ thin film can be observed on the entire surface of b-Si needles shown in Fig. 3b.

**Fig. 4:**
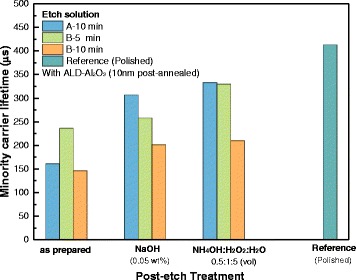
The effective minority carrier lifetime of samples etched by solutions A and B with different post-etch treatments, using polished wafer as a reference. Samples were passivated with double-side ALD-deposited Al_2_O_3_ thin film, post-annealed

Figure 4 shows the effective minority lifetime for all samples passivated by ALD-deposited Al_2_O_3_ thin film and post-annealing. Annealing process affects a lot on the passivation quality of Al_2_O_3_ thin film. The effective minority carrier lifetime of polished wafer was only 10 μs before annealing, and increased to 413 μs after. For the group of as-prepared samples, etching depth determines the values of the effective minority carrier lifetime. Sample etched by solution B for 5 min gives a relatively shallower etching depth and thus gains the highest value in this group. Post-etch treatment increases the effective minority carrier lifetime. For example, for samples etched by solution A for 10 min, the effective minority carrier lifetime was increased from 161 μs before to 307 and 333 μs after post-etch treatment of NaOH and NH_4_OH/H_2_O_2_/H_2_O, respectively. Regardless of the concentration of etching solution, the effective minority carrier lifetime is higher in NH_4_OH/H_2_O_2_/H_2_O post-etch-treated samples than in NaOH post-etch-treated samples. Considering samples with high values of effective minority carrier lifetime were achieved through NH_4_OH/H_2_O_2_/H_2_O post-etch treatment, NH_4_OH content in mixed solution will be optimized.

### Effect of the NH_4_OH Content in Post-etch Treatment

An NH_4_OH/H_2_O_2_/H_2_O mixed solution having three different levels of volume ratio was used, in which NH_4_OH content was adjusted in 0.2:1:5, 0.5:1:5, and 1:1:5. During the preparation of these samples, the Ag^+^ concentration in the AgNO_3_/HF mixed solution was increased in order to strengthen the degree of etching to further reduce the reflectance. The weighted reflectance of as-prepared sample decreased from 8.4% of the original AgNO_3_/HF mixed solution to 6.8% of the new.

**Fig. 5:**
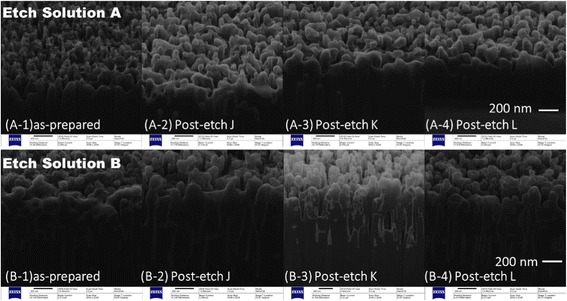
SEM picture of samples etched by solutions **a** and **b** (10 min) with different post-etch treatments

Figure 5 shows the SEM images of samples before and after post-etch treatment. The size of the nanostructure of samples etched by solution A for 10 min is 200 nm, while that of solution B is 500 nm. Post-etch treatment has a great influence on surface morphology. Without the post-etch treatment, the as-prepared samples have rough surfaces with many sharp burrs. After the post-etch treatment, samples’ surfaces become smooth and bright. And the size of the nanostructure is not obviously changed through post-etch treatment. Nevertheless, changing the NH_4_OH content in post-etch solution has no significant effect on surface morphology.

**Fig. 6:**
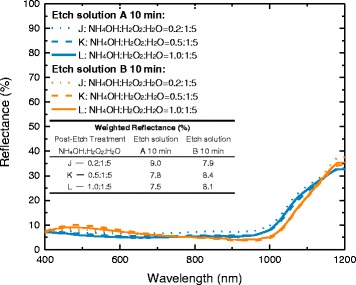
Reflectance of samples etched by solutions A and B with different post-etch treatments

The reflectance spectrums of samples as well as the AM1.5G-weighted reflectance are shown in Fig. 6. Although the reflectance spectrum has some changes in the shape of lines, the effect of NH_4_OH content on the anti-reflection quality of b-Si on solar cells is very small, because the AM1.5G-weighted reflectance is almost unchanged at different NH_4_OH contents.

**Fig. 7:**
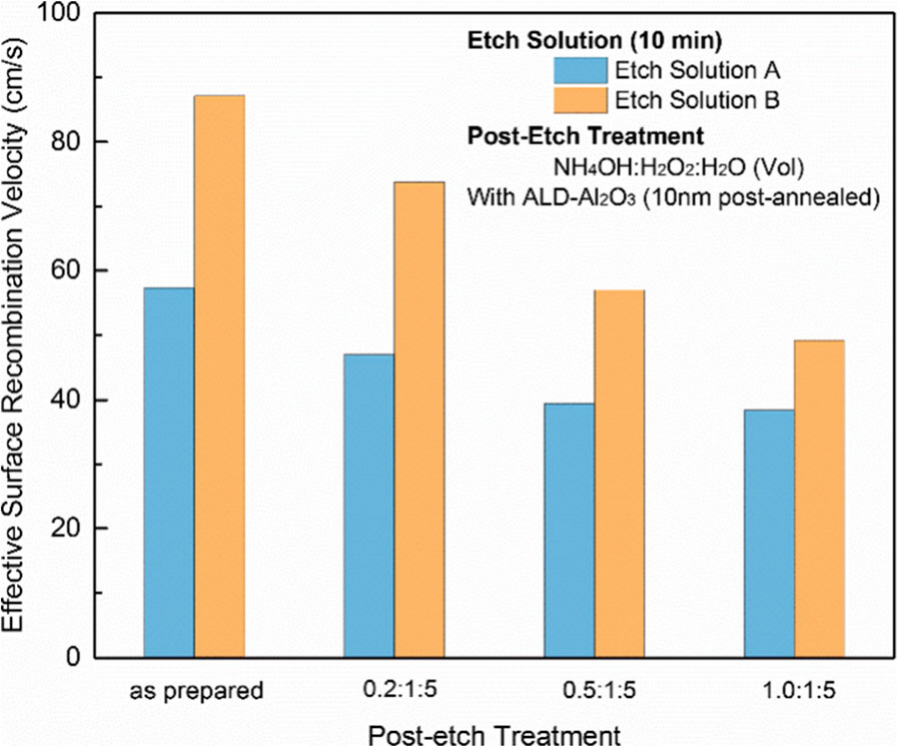
The effective surface recombination velocity of samples etched by solutions A and B with different post-etch treatments. Samples were passivated with double-side ALD-deposited Al_2_O_3_ thin film, post-annealed

However, a decrease of effective surface recombination velocity is observed as a function of the NH_4_OH content in mixed solution shown in Fig. 7. For black silicon surfaces, the total recombination caused by nanostructure can be reduced to a flat plane just below the nanostructure using an effective surface recombination velocity [24]. And the value of the effective surface recombination velocity can be calculated from the measured effective minority carrier lifetime according to Equation (2):

2 $$ \frac{1}{\tau_{\mathrm{eff}}}=\frac{1}{\tau_{\mathrm{b}}}+\frac{S_{\mathrm{front}}+{S}_{\mathrm{Back}}}{W} $$

where *τ*_eff_ is the measured effective minority carrier lifetime, *τ*_b_ is the carrier bulk lifetime, *W* is the wafer thickness, *S*_front_ and *S*_Back_ are the effective surface recombination velocity of front surface and back surface, respectively. Assuming an infinite carrier bulk lifetime [25], the effective surface recombination velocity can be calculated rather easily. The corresponding surface recombination velocity for polished reference is *S*_front_ = *S*_Back_ = *S*_eff_ = 22 cm/s. In the case of black silicon, only the front surface has nanostructure, and the back surface is polished. Equation (2) and infinite carrier bulk lifetime assumption are still valid, and the effective surface recombination velocity of back surface can be taken as the value of polished reference. Therefore, the sample that was etched by solution A and post-etch-treated by NH_4_OH/H_2_O_2_/H_2_O (volume ratio 1.0:1:5) mixed solution achieves the minimum effective surface recombination velocity of 38 cm/s. This value is smaller than the 56 cm/s that Oh et al. [26] reported for their 18.2% efficient metal etched b-Si solar cell.

Post-etch treatment smoothes the surface, and increasing NH_4_OH content in mixed solution will enhance the smoothness. Technically, this process will usually lead to increased reflectance. But because of the deep structure of nanowires, the effect of increasing NH_4_OH content on the reflectance is not obvious. However, increasing NH_4_OH content can greatly improve the effective minority carrier lifetime. This means increasing the NH_4_OH content in mixed solution can improve the electrical properties of b-Si without sacrificing the optical properties.

### Effect of the Illumination in Etching Process

In order to further improve the reaction rate of the etching process, bulb illumination was added on the basis of indoor lighting during MCCE process in the HF/H_2_O_2_ mixed solution. According to the etching principle of MCCE method, the number of holes injected into the valence band of Si has a great influence on the etching rate. In indoor lighting conditions, because the intensity of illumination is low, so the etching rate depends on the concentration of silver ions acting as a catalyst and H_2_O_2_ acting as an oxidant [8]. However, adding a bulb illumination on the basis of indoor lighting, because the intensity of illumination is high enough to obtain a sufficient number of minority carriers, so the etching rate is accelerated by photo-generated carriers.

**Fig. 8:**
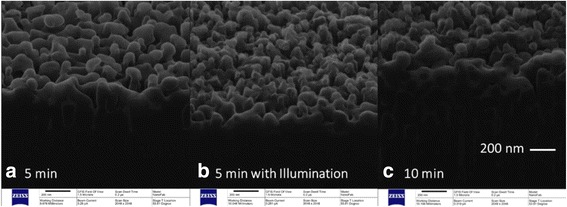
Cross-sectional SEM images of samples etched for 5 and 10 min, respectively, under the conditions of indoor lighting illumination and bulb illumination. Samples were etched by solution B and without any post-etch treatment after Ag^+^ removal. **a** Sample etched for 5 min under indoor lighting. **b** Sample etched for 5 min under bulb illumination. **c** Sample etched for 10 min under indoor lighting

Figure 8 compares the cross-sectional SEM images of samples etched for 5 and 10 min, respectively, under the conditions of indoor lighting illumination and bulb illumination. Samples were etched by solution B and without any post-etch treatment after Ag^+^ removal. In the absence of bulb illumination, comparing samples etched for 5 and 10 min in Fig. 8a, c, respectively, the etching depth is increased by extending the etching time. However, when the etching time is 5 min, comparing samples etched without and with bulb illumination in Fig. 8a, b, respectively, the addition of bulb illumination does not increase the etching depth in the same etching time, but makes the nanostructure more compact and uniform. That is, illumination-enhanced wet chemical etching makes the size of nanostructures smaller and the surface of b-Si denser.

**Fig. 9:**
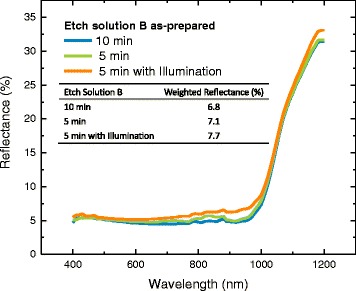
Reflectance of samples etched for 5 min and 10 min, respectively, under the conditions of indoor lighting illumination and bulb illumination. Samples were etched by solution B and without any post-etch treatment after Ag^+^ removal

**Fig. 10:**
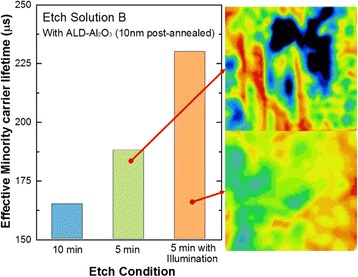
The effective minority carrier lifetime of samples etched for 5 min and 10 min, respectively, under the conditions of indoor lighting illumination and bulb illumination. Samples were etched by solution B and without any post-etch treatment after Ag^+^ removal and then samples were passivated with double-side ALD-deposited Al_2_O_3_ thin film, post-annealed

Being different with the effect on the surface morphology, adding bulb illumination during the etching process has no obvious influence on samples’ reflectance, as shown in Fig. 9. The weighted reflectance of the three samples is about 7%, without obvious difference. However, the addition of bulb illumination can greatly improve the effective minority carrier lifetime, which increased from 188 μs under indoor lighting illumination to 230 μs under bulb illumination, as shown in Fig. 10. Moreover, the uniformity of effective minority carrier lifetime on the whole surface was also improved a lot. This is consistent with the fact that the addition of bulb illumination makes the surface morphology more uniform. In a word, adding bulb illumination in etching process changes little on reflection but increases the effective minority carrier lifetime as well as the uniformity.

## Conclusions

Black silicon was fabricated on n-type solar grade silicon wafers using an optimized MCCE method and passivated using an ALD-deposited Al_2_O_3_ thin film, post-annealed. Spray method for the deposition of silver ions results in a single-side etching without using masks. After post-etch treatment, b-Si exhibits a low reflectance of 6–10%. The use of NH_4_OH/H_2_O_2_/H_2_O in place of NaOH as post-etch treatment to smooth the surface not only achieves higher effective minority carrier lifetime of 333 μs but also eliminates the need for the Na^+^ removal step. At the same time, increasing the NH_4_OH content in the post-etch solution can improve the effective minority carrier lifetime without significantly increasing the reflectance. Also, an effective surface recombination velocity of 38 cm/s is achieved on sample post-etch treated by NH_4_OH/H_2_O_2_/H_2_O (volume ratio 1.0:1:5) mixed solution, while the value of polished reference is 22 cm/s. Moreover, by adding illumination during the etching process, the etching rate can be increased, and a finer uniform surface can be obtained, resulting in an improvement in both the numerical value and the uniformity of the effective minority carrier lifetime.
